# HIV therapeutic vaccine enhances non-exhausted CD4^+^ T cells in a randomised phase 2 trial

**DOI:** 10.1038/s41541-019-0117-5

**Published:** 2019-06-03

**Authors:** Vincent Vieillard, Béhazine Combadière, Roland Tubiana, Odile Launay, Gilles Pialoux, Laurent Cotte, Pierre-Marie Girard, Anne Simon, Yasmine Dudoit, Jacques Reynes, Jürgen Rockstroh, Felipe Garcia, Jose Gatell, Alain Devidas, Yazdan Yazdanpanah, Laurence Weiss, Gerd Fätkenheuer, Brigitte Autran, Delphine Joyeux, Shahin Gharakhanian, Patrice Debré, Christine Katlama

**Affiliations:** 1 Sorbonne Université, Inserm, CNRS, Centre d’Immunologie et des Maladies Infectieuses (CIMI-Paris), Paris, France; 2 AP-HP Pitié-Salpêtrière, Paris, France; 3 Sorbonne Université, UPMC Univ Paris 06, Inserm, Pierre Louis Institute of Epidemiology and Public Health, Paris, France; 4 AP-HP Cochin, Paris, France; 5 AP-HP Tenon, Paris, France; 6 Hospices Civils de Lyon, Hôpital de la Croix-Rousse, Lyon, France; 7 AP-HP Saint-Antoine, Paris, France; 8 Hôpital Gui de Chauliac, Montpellier, France; 9 Unité Mixte Internationale “TransVIHMI”, IRD UMI233, Inserm U1175, Université de Montpellier, Montpellier, France; 10 Universität Klinikum Bonn, Bonn, Germany; 11 Hospital Clínic, Barcelona, Spain; 12 ViiV Healthcare, Brentford, UK; 13 Centre hospitalier Sud Francilien, Corbeil-Essonne, France; 14 AP-HP Bichat, Paris, France; 15 AP-HP Hôpital Européen Georges Pompidou, Paris, France; 16 Université Paris Descartes, Sorbonne Paris-Cité; Inserm, Paris, France; 17 Department 1 for Internal Medicine, University Hospital of Cologne, Cologne, Germany; 18 German Centre for Infection Research, Partner Site Bonn-Cologne, Cologne, Germany; 19 Minka Therapeutics, Paris, France; 20 Pharmaceutical Medicine & Infectious Diseases, CIC: Cambridge Innovation Center, Cambridge, MA USA

**Keywords:** Viral infection, Peptide vaccines, Viral infection

## Abstract

VAC-3S is a therapeutic vaccine comprising a highly conserved HIV-gp41 motif coupled with the CRM197 carrier protein. High levels of anti-3S antibodies (Abs) have been associated with improved protection of CD4^+^ T-cell survival. A previous phase 1 study demonstrated the safety of VAC-3S. This multicentre, randomised, double-blind, placebo-controlled phase 2 clinical trial enroled between January 2014 and March 2015 HIV-1-infected patients under ART with plasma HIV RNA levels below 50 copies/mL and CD4 counts between 200 and 500 cells/μL. Participants were immunised with 16, 32, or 64 μg of VAC-3S, and compared to placebo. The primary outcome was immunogenicity assessed by changes from baseline of anti-3S Abs levels at week 12. Secondary outcomes included adverse events and the course of plasma HIV RNA level, CD4 count, CD4/CD8 ratio, inflammation and immune checkpoints from week 0 to week 48. Vaccination was well tolerated with no serious adverse events and induced a significant increase in anti-3S Ab response in vaccinated patients (*p* < 0.0001), compared to placebo. In high responders, the robust increased of CD4 count was associated with a significant and sustained reduction of PD-1 expression on CD4^+^ T cells through week 48 (variance *p* = 0.0017). PD-1 expression was correlated with level of anti-3S Abs (*p* = 0.0092, *r* = −0.68) and expression of NKp44L (*p* < 0.0001; *r* = 0.54) in CD4^+^ T cells. Our findings regarding the increase of non-exhausted CD4^+^ T cells have potentially important application in personalised HIV vaccination for HIV-infected patients with high level of PD-1 to improve their T-cell immune function.

## Introduction

Despite control of viral replication leading to improvement in survival and prevention of transmission, HIV infection remains characterised by persistent immune dysfunction and inflammation that account at least partly for the increased risk of non-AIDS-related morbidities and a wide range of complications.^1–3^ Although it can fully suppress viral replication, combined antiretroviral therapy (cART) cannot effectively reconstitute CD4^+^ T cells to pre-infection levels in every patient.^4,5^ Indeed 40% of patients starting treatment with a CD4 count below 200 cells/μL failed to achieve more than of 500 cells/μL after 10 years of cART.^6^ Although the mechanisms remain unclear, evidence suggests that the initiation of treatment during primary infection or early infection rather than later during chronic infection significantly improves the prospects for enhanced immune recovery.^7^ World-wide, still is a large proportion of HIV infections diagnosed at late stage and exposed to slow or incomplete CD4^+^ T-cell recovery. The persistence of CD4^+^ T-cell immunologic dysfunction seems to be associated with several different mechanisms, including increased cell turnover, exhaustion, and altered homoeostatic responses. Furthermore, CD4 counts have been successfully expanded after IL-2 treatment in combination with ART in two large studies (SILCAAT and ESPRIT), with increases of 53 and 159 cells/year on average,^8^ and by IL-7 treatment with ART in a smaller study.^9^ However, neither strategy has been implemented due to a high toxicity rate mediated by Treg,^10^ together with a lack of clinical benefit by IL-2 and the negative effects on reservoirs by IL-7. Altogether, these data suggest that, in order to have clinical impact, the normalisation of the CD4 count is not sufficient without full restoration of CD4^+^ T-cell functional competence.^11,12^

We have proposed that CD4^+^ T-cell depletion might be partly mediated by deleterious effects of NK cells,^13^ as reported in several infections such as hantavirus infection, in which the destruction of uninfected endothelial cells by activated NK cells partially explains the detrimental increase in vascular permeability.^14,15^ In HIV-1, we have shown that a highly specific and conserved motif located in gp41, named 3S, is able to specifically induce the cell-surface expression of NKp44L, a stress ligand of the NKp44 activating NK-cell receptor.^16,17^ We further reported a correlation between a high level of anti-3S antibodies (Abs) in untreated HIV-infected patients and both slower disease progression and CD4^+^ T-cell depletion.^18,19^ Hypothesising that anti-3S antibody could block NKp44L expression and thus inhibit NK cell-mediated CD4^+^ T-cell depletion, we immunised simian HIV (SHIV)-infected macaques with a 3S-based vaccine. An increased anti-3S Abs together with protection of CD4^+^ T cells from death and decreased immune activation and inflammation in both peripheral and secondary lymphoid tissues was observed.^20,21^ We thus developed the VAC-3S therapeutic vaccine, comprising the 3S peptide coupled with the CRM197 carrier protein formulated in an aluminium salt adjuvant. We demonstrated it to be both safe and immunogenic in a First-In-Human study at doses of 10 and 20 µg in HIV-1 infected patients under cART with suppressed viremia and CD4 count ≥200 cells per mm^3^ (ClinicalTrials.gov, #NCT01549119).^22^

In the present study results of IPROTECT1, a randomised, double-blind, placebo-controlled phase II study are shown. The trial’s overall objectives were to assess the safety and the immunogenicity and to optimise the dose and dosing regimen of VAC-3S. The secondary end point was to measure the effect of vaccination on changes in CD4 count, CD4/CD8 ratio, and inflammation markers; and, in a post-hoc analysis, to assess these effects after categorising patients according to the anti-3S Ab responses.

## Results

### Study participants

**Fig. 1:**
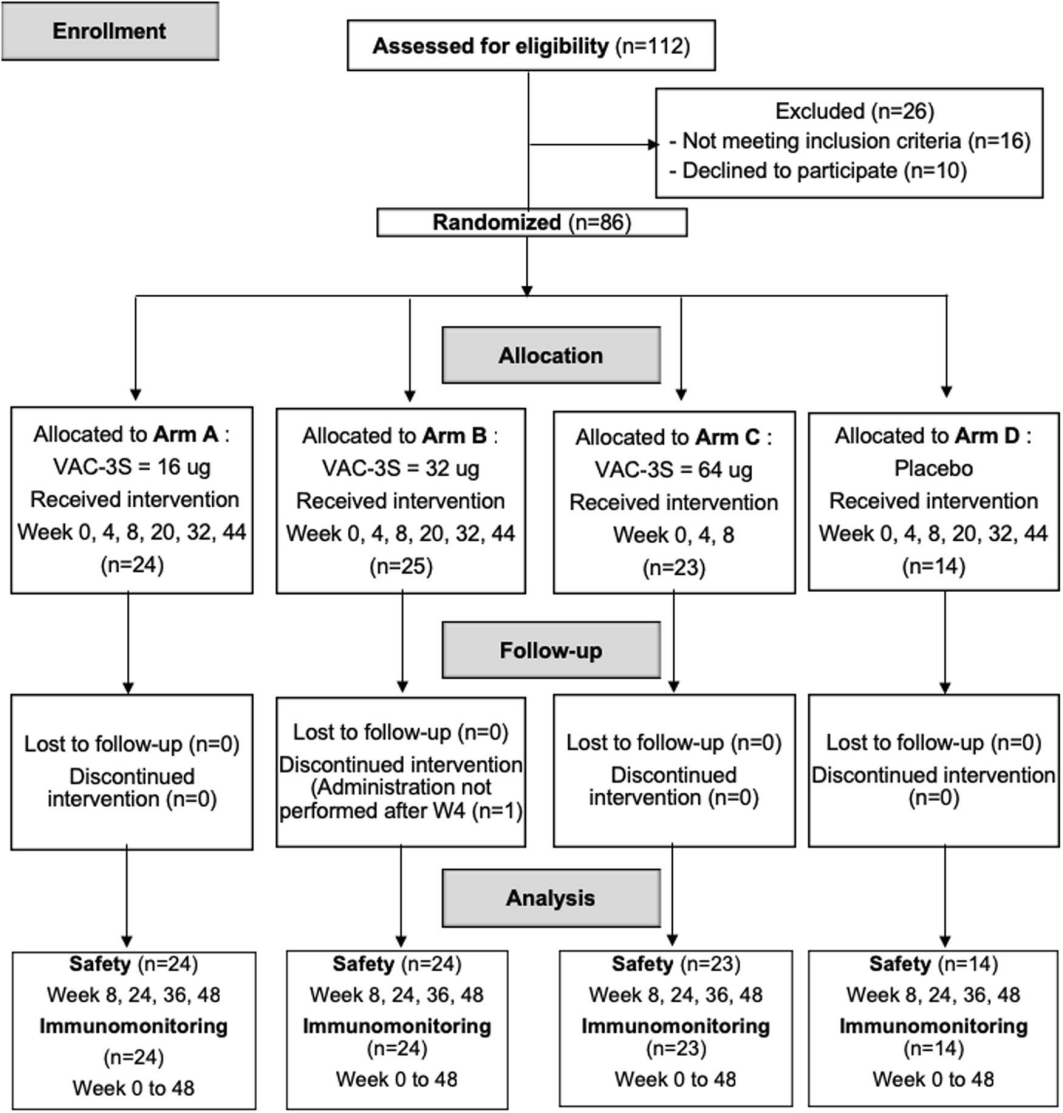
Study flow flow-chart of enrolment, allocation and analysis process in HIV vaccine Phase 2, double-blind, dose-ranging study

**Fig. 2:**
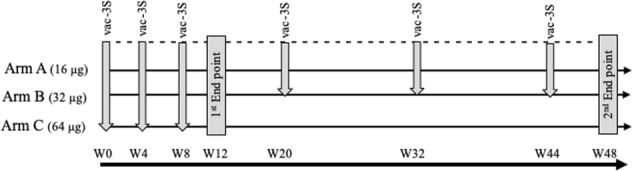
Study design. The six immunisations with VAC-3S between week 0 and week 44 are indicated by arrows, and the first and second end points by closed bars

Between January 2014 and March 2015, 112 HIV-1-infected patients under ART were screened and 86 enroled. The flow chart specifies the reasons for non-enrolment. There was one patient lost to follow-up in arm B, 85 patients (98.8%) completed the study (Fig. 1). They were randomised as follows: arm (A) VAC-3S dose 16 μg (*n* = 24), arm (B) 32 μg (*n* = 25), arm (C) 64 μg (*n* = 23), and arm (D) placebo (*n* = 10) (Fig. 2) and received three intramuscular injections of placebo or 16 or 32 μg VAC-3S at 4 week-intervals until week 8, followed by three more injections at weeks 20, 32, and 44, whereas the 64-μg dose was injected only at weeks 0, 4, and 8 (Fig. 1). Patients’ characteristics were well balanced between groups at baseline (Table 1): patients were mostly male (79.1%), with a median age of 48 years (IQR 42–54). The median time since HIV diagnosis was 11.9 years (IQR 5.5–22.1) with a CD4 nadir of 159 cells per mm^3^ (IQR 90–213), a baseline CD4 count of 364 cells per mm^3^ (IQR 300–426) and a baseline total cell-associated HIV DNA level of 738 copies (384–1088) per 10^6^ PBMCs.

**Table 1:** Baseline characteristics

	Vaccine groups						Placebo group		All
	16 µg (*n* = 24)		32 µg (*n* = 25)		64 µg (*n* = 23)		Placebo (*n* = 14)		
	CD4 < 350	CD4 ≥ 350	CD4 < 350	CD4 ≥ 350	CD4 < 350	CD4 ≥ 350	CD4 < 350	CD4 ≥ 350	
*N*	14	10	9	16	8	15	6	8	86
% Men	10 (71.4)	9 (90.0)	7 (77.8)	12 (75.3)	7 (87.5)	14 (93.3)	4 (66.7)	5 (62.5)	68 (79.1)
White race, %	7 (50.0)	7 (70.0)	5 (55.6)	8 (50.0)	6 (75.0)	11 (73.3)	4 (66.7)	4 (50.0)	52 (60.5)
Age (years)	44 (39–50)	48 (43–51)	48 (42–51)	48 (42–51)	54 (42–55)	47 (43–54)	55 (46–56)	50 (43–53)	48 (42–54)
HIV duration (years)	7.5 (5.0–13.1)	16.6 (6.4–24.6)	10.1 (5.5–15.9)	10.6 (7.9–23.3)	7.1 (3.7–19.4)	15.3 (10.9–20.6)	25.7 (5.7–27.7)	12.4 (5.1–20.6)	11.9 (5.5–22.1)
cART duration (years)	7.4 (4.4–26.1)	13.4 (2.3–18.5)	8.2 (4.1–21.7)	9.3 (6.9–16.1)	6.3 (3.2–13.3)	12.7 (7.4–19.6)	8.6 (3.2–23.1)	10.2 (4.9–24.9)	9.3 (5.1–17.8)
Nadir CD4 count (c/mm^3^)	133 (75–170)	170 (88–232)	160 (140–171)	168 (81–238)	124 (86–172)	199 (106–246)	85 (64–122)	244 (166–305)	159 (90–213)
CD4 count (c/mm^3^)	269 (249–301)	444 (412–481)	280 (267–307)	415 (396–435)	306 (286–327)	410 (364–464)	289 (257–33)	415 (369–434)	364 (300–426)
% CD4	22.9 (17.0–28.5)	29.8 (27.5–37.2)	24.2 (22.5–28.5)	27.0 (22.9–33.0)	26.2 (18.0–36.6)	25.5 (21.5–32.0)	22.5 (21.5–31.0)	30.8 (27.8–35.0)	27.1 (19.8–32.2)
CD4/CD8 ratio	0.50 (035–0.77)	0.85 (0.58–1.07)	0.58 (0.47–0.90)	0.65 (0.54–1.04)	0.47 (0.40–0.91)	0.59 (0.36–0.83)	0.46 (0.37–0.65)	0.78 (0.66–0.93)	0.62 (0.40–0.91)
HIV DNA (copies/10^6^ PBMC)	841 (603–1122)	602 (398–872)	649 (306–1004)	739 (422–929)	786 (384–1113)	691 (259–1463)	1403 (426–2369)	347 (191–985)	738 (384–1088)

No statistically significant difference has been observed across study arms for any parameter. Data are in (%) or median (IQR)

### Safety of VAC-3S

After vaccination, local and systemic reactions occurred with similar frequency and severity in the vaccine and placebo recipients. Most patients (69%) experienced mild local reactions at the site of immunisation. Unrelated severe adverse events were recorded in one vaccinated patient (blood HIV RNA increase) and one placebo patient (acute hepatitis) (Supplementary Tables 1 and 2). Overall, the vaccine formulation was considered safe. There was no discontinuation from the vaccine schedule due to adverse reactions.

### Immunogenicity of VAC-3S

At week 12 (primary endpoint), the proportion of vaccine responders was 45.8% (*p* = 0.0026), 62.5% (*p* = 0.0002), and 47.8% (*p* = 0.0020) in arms A, B, and C, respectively, as compared with 0% in the placebo group. The level of anti-3S Abs in immunised patients increased significantly in all the vaccine arms (*p* = 0.0026 for arm A; *p* = 0.0002 for arm B, and *p* = 0.0020 for arm C), compared with placebo, regardless of CD4 count at inclusion (Supplementary Table 3).

**Fig. 3:**
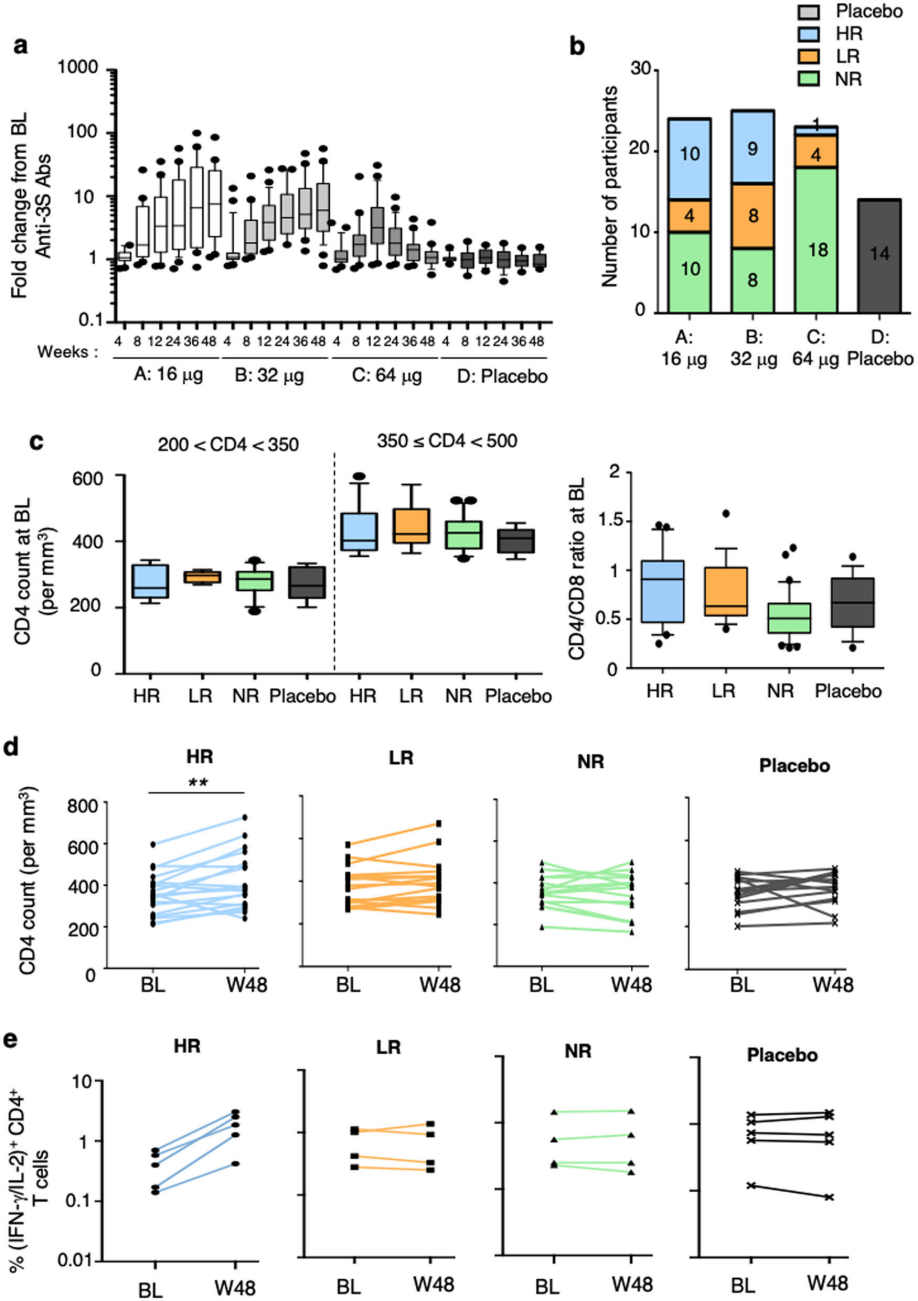
Immunogenicity and CD4 counts over time in patients vaccinated with VAC-3S. Fold changes from baseline (BL) of anti-3S Ab levels in serum samples from patients at different time points (week 4–week 48) in the four study arms: A 16 μg, B 32 μg, C 64 µg of VAC-3S vaccine, and D placebo in **a**. Classification of subjects as high (HR, ≥ 10-fold change), low (LR, > 4-fold change), and non-responders (NR, ≤4-fold change) in terms of their anti-3S response at week 48 compared to baseline (BL) in **b**. CD4 counts in patients with 350 < CD4 < 500 cells and CD4/CD8 ratios at baseline in high (HR), low (LR), and non-responders (NR), compared to placebo in **c**. D Course of CD4 count per mm^3^ between baseline (BL) and week 48 (W48) in high (HR), low (LR), and non-responders (NR), and placebo patients in **d**. **p < 0.001 (Wilcoxon matched-pairs test). Frequency of CD4^+^ T cells expressing IFN-γ and/or IL-2 following PPD stimulation is shown in **e**. CD4^+^ T cell responses were assessed after peripheral blood mononuclear cells (PBMC) stimulation with PPD (10 μg/mL) at baseline (BL) and week 48 (W48) in high (HR, *n* = 5), low (LR, *n* = 4), and non-responders (NR, *n* = 4), compared to placebo (*n* = 5). All values have been corrected for isotype controls and production in the absence of recall Ag. Control cells stimulated with *Staphylococcus* enterotoxin B (2 μg/mL; positive control) or media alone (negative control) are not shown

There was a continuous significant increase in anti-3S Abs levels until week 48 in both treatment groups receiving a total of six injections of VAC-3S (arm A *p* = 0.0001 and arm B *p* < 0.0001) but not in patients from arm C who received only three injections (*p* = 0.2315). It is however important to note the marked heterogeneity in the quantitative anti-3S responses in all groups (1 <fold-change>100; Fig. 3a). While the anti-3S Ab levels remained significantly higher at week 48 than at baseline, there was no change in terms of CD4^+^ and CD8^+^ T-cell proportions or absolute values, CD4/CD8 ratios, total HIV DNA and RNA levels, or inflammatory markers (IL-6, CRP, DDIMER, and TNFAR2) (Supplementary Table 4).

### Enhanced quantity and quality of CD4^+^ T cells after VAC-3S vaccination

**Fig. 4:**
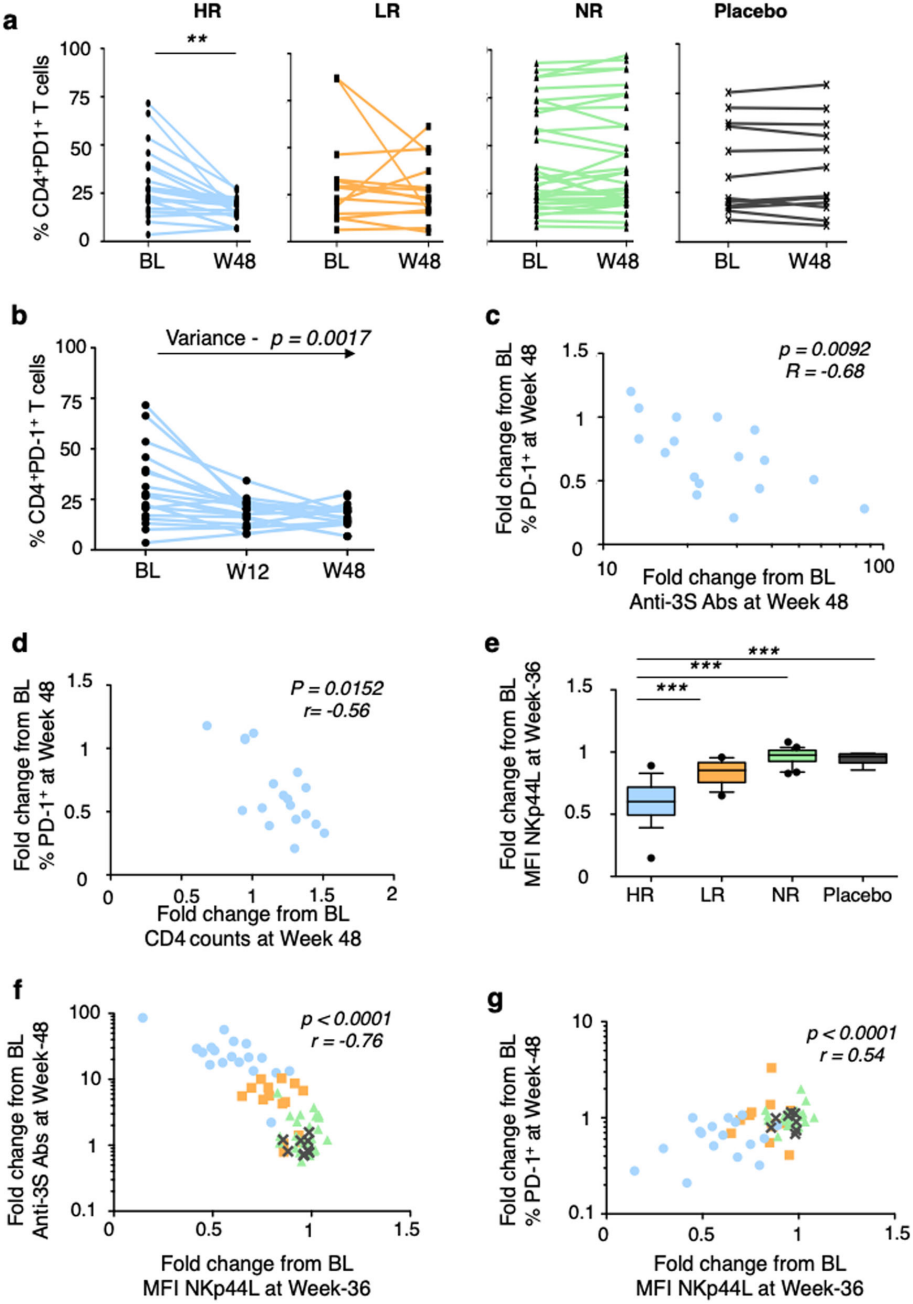
Immunomodulation in patients vaccinated with VAC-3S Course of PD-1 frequency on CD4^+^ T cells between baseline (BL) and week 48 (W48) in high (HR), low (LR), and non-responders (NR), and placebo patients in **a**. **p < 0·001 (Wilcoxon matched-pairs test). Kinetics of PD-1 expression on CD4^+^ T cells from baseline (BL) to week 12 (W12) to W48 in each high responder (HR). Significant values of the variance are indicated in **b**. Correlation of changes from baseline (BL) to week 48 in high responders between the frequency of PD-1 expression on CD4^+^ T cells and anti-3S production in **c**, and the CD4 count in **d**. Fold changes from baseline (BL) to week 36 of NKp44L expression, expressed in mean of fluorescence intensity (MFI) on CD4^+^ T cells treated by serum samples from high (HR), low (LR), and non-responders (NR), compared to placebo in **e**. ***p < 0·0001 (Mann–Whitney test). Correlation of fold changes from baseline (BL) between NKp44L expression and anti-3S production. High responders (blue circles), low responders (orange squares), non-responders (green triangles), and placebo (grey cross) in **f**. Correlation was evaluated with a Spearman rank correlation coefficient test. Correlation of changes from baseline (BL) between NKp44L expression and the frequency of PD-1 expression on CD4^+^ T cells in **g**. High responders (blue circles), low responders (orange squares), non-responders (green triangles), and placebo (grey cross). Correlation was evaluated with a Spearman rank correlation coefficient test

To parse the heterogeneity of the quantitative anti-3S response, compared with immune responses against the carrier (anti-CRM197 Abs, Supplementary Fig. 1a), we performed a post-hoc analysis considering the stratification of vaccinated patients into high, low, and non-responders, previously used for the evaluation of other vaccines,^23^ according to their anti-3S Ab fold-increase (≥10, >4, and ≤4, respectively) between baseline and week 48. Overall 27.8% were high responders, 22.2% low responders, and 50.0% non-responders (Fig. 3b). At week 48, median anti-3S Ab production was 183 U/mL (95% CI of the mean 160–360) in high responders, 52 U/mL (95% CI 35–82) in low responders, and 13 U/mL (95% CI 11–48) in non-responders, compared to 9 U/mL (95% CI 5–24) in placebo (Supplementary Fig. 2a). There was no difference between those 4 groups (high, low, and non-responders and placebo) in terms of known duration of HIV infection, duration of viral suppression under ART, as well as the CD4^+^ T-cell nadir, HIV-DNA, age, CD4 count, and CD4/CD8 ratio at the baseline (Table 1; Fig. 3c, Supplementary Fig. 3). Furthermore, at baseline, there was no significant difference between groups in term of CD4^+^ T cell responses to a super-antigen (SEB) and a recall antigen (PPB) (Supplementary Fig. 2b). Similar data were observed for the level of anti-CRM Abs (Supplementary Fig. 1b); the CRM197, used as carrier in the VAC-3S vaccine, is also considered as a recall antigen in patients vaccinated against the diphtheria toxin. Importantly, a significant median increase of 60 CD4^+^ T-cell /mm^3^ was observed in high responders (*p* = 0.0029) during the first 48 weeks, contrasting with no change in low and non-responders or in the placebo group (Fig. 3d), while a very transient decreased of CD4^+^ T cell count was observed during the first endpoint (Supplementary Table 4), as previously described.^24,25^ The increased of CD4 count in high responders was associated with a 3.0 to 7.2-fold increase of the CD4^+^ T cell response to tuberculin purified protein derivate (PPD) in the five tested patients during the first 48 weeks, whereas no important change was observed in tested patients of the other groups (Fig. 3e). while the proportion of activated cells was similar in the different groups (Supplementary Fig. 4), we also observed a significant decrease in PD-1 expression on CD4^+^ T cells at the two time points tested in high responders, but not in the other vaccine arms or the placebo arm (*p* = 0.0021 at week 48) (Fig. 4a, Supplementary Fig. 5). Modulation of PD-1 expression was thus time-dependent (variance *p* = 0.0017, Fig. 4b) and inversely correlated with change from baseline of anti-3S Abs levels (*p* = 0.0092, *r* = −0.68) and of CD4 counts (*p* = 0.0152, *r* = −0.56) in high responders (Fig. 4c, d). Decreased PD-1 expression on CD4^+^ T cells among high responders was also associated with an increase of sDDP4 activity in serum (*p* = 0.014 at week 24), a systemic surrogate marker that strikingly predicts time to AIDS,^26–28^ as compared to the other groups or the placebo (Supplementary Fig. 5).

Finally, we assessed the functional effect of anti-3S Abs from VAC-3S vaccinated patients by measuring their capacity to inhibit NKp44L expression on CD4^+^ T cells, as previously performed in two pre-clinical macaque models.^20,21^ Figure 4e shows a significant inhibition of NKp44L expression in the presence of serum samples from high responders (*p* < 0.0001), compared with all other groups. In contrast, NKp44L expression was inversely correlated with change from baseline of anti-3S Abs (*p* < 0.0001, *r* = −0.76, Fig. 4f) and sDDP4 activity (*p* = 0.0002, *r* = −0.48; Supplementary Fig. 5). As expected, the course of NKp44L expression was positively correlated with changes from baseline of PD-1 expression on CD4^+^ T cells (*p* < 0.0001, *r* = 0.54; Fig. 4g). Of note, kinetic studies for the level of blood HIV DNA between baseline, week 12 and week 48 revealed no significant differences among study groups (Supplementary Fig. 5), suggesting that the anti-3S vaccine had no direct effect on the viral reservoir, but preserved and/or improved the quality of the CD4^+^ T cells by decreasing PD-1 and NKp44L expression.

## Discussion

This randomised placebo-controlled study shows three major results of immunisation with VAC-3S of chronically HIV-infected patients with incomplete CD4 cell reconstitution. First, the vaccine induced 3S Abs in 45.8–62.5% of patients, and the immunisation rate was higher with six injections of 16-µg or 32-µg VAC-3S regimens than with three injection of 64-µg regimen, suggesting that 3 injections, even if at a higher dose, is not sufficient to maintain a significant anti-3S response. Second, confirming results from the clinical phase 1 study, the vaccine was safe with mainly limited local reactions and no systemic side effects inducing discontinuation of vaccine immunisations. Third, a high level of VAC-3S Abs led to a significant increase in CD4^+^ T cells of about 60 cells/mm^3^ per 48 weeks in high responders in association with a higher CD4^+^ T cell response to recall antigen PPD in tested patients. CD4^+^ T cell increases is also correlated with a beneficial effect on their immunomodulatory markers, including down-modulation of PD-1 and NKp44L expression on CD4^+^ T cells, as well as increased sDDP4 activity in serum, regardless of age, HIV duration, nadir CD4, HIV-DNA load, and CD4^+^ T cell response at the baseline.

These data are broadly consistent with the preclinical data obtained in SHIV-infected macaques, where VAC-3S vaccination was associated with a significant preservation of peripheral CD4^+^ T cells, related to down-modulation of markers linked to inflammation, cell activation, and NK-mediated apoptosis in lymphoid tissues.^20,21^

In the clinical study presented here, high responders experienced a median production of anti-3S Abs over 180 U/mL at week 48, a titre close to that observed in the 3S-vaccinated macaques (about 200 U/mL) together with a substantial increase in CD4 counts,^20^ whereas persistent low CD4 counts have been associated with non-AIDS related events.^11^

Importantly, the CD4^+^ T-cell increase in high responders observed in this study was clearly associated with changes in immunomodulatory markers on these cells, including down-modulation of PD-1 expression and increased serum sDDP4 activity, regardless of age, HIV duration, nadir CD4, or HIV-DNA load. PD-1 is well known to be upregulated on both total CD4^+^ and CD8^+^ T cells in untreated HIV infection, but is partially normalised with antiretroviral therapy. Furthermore its expression increases on HIV-specific T cells and may contribute to HIV persistence,^29–31^ while its in vitro inhibition increases HIV-specific T-cell functions and CD4^+^ T-cell proliferation.^32^ Additionally, PD-1^+^ CD4^+^ T cells were proposed reservoir for latent HIV-1 in ART-suppressed patients,^33^ and in vivo blockade of PD-1 in HIV-infected patients treated with PD-1 antagonists for cancer led in one case to a substantial reduction of HIV reservoirs.^34^ Although the number of high responder patients was small, the significant decrease in PD-1 expression in patients who produced the highest levels of anti-3S Abs suggests that VAC-3S vaccination might induce a preferential increase of non-exhausted CD4^+^ T cells in HIV-infected patients. Similarly, we also report that 3S-vaccinated patients showed significantly higher sDPP4 activity; this immunomodulatory marker plays a key role in activation and proliferation of T lymphocytes, decreases strongly in HIV-1 patients, and is not restored under cART.^26–28^ Moreover, it is associated with a significant reduction in NKp44L expression, as previously reported in the peripheral blood and lymphoid organs of SHIV_162P3_-infected macaques vaccinated with the 3S peptide.^20,21^ It is however important to note that NKp44L is only expressed on bystander non-infected cells and not on HIV-infected cells, by a HIV Nef-dependent pathway.^35,36^ This suggests that HIV-infected CD4^+^ T cells should not be a main target for NK cells, and by extrapolation, therapeutic vaccination with VAC-3S should not have a major impact on the HIV reservoir and its clearance after reactivation of the provirus.

To the best of our knowledge, this study is the first clinical trial of a therapeutic vaccination in which CD4 counts increased at the same time that the level of PD-1 linked to key effector players in HIV pathogenesis fell. We can therefore hypothesise that effective vaccination by VAC-3S could possibly modulate the immune checkpoint expression and may offer a valuable tool for restoring immune function in virally suppressed individuals with incomplete CD4 cell reconstitution. We acknowledge that these elements are driven from a post hoc analysis and should be confirmed as prespecified evaluation criteria in future clinical studies.

Another limitation to the current VAC-3S formulation was the small proportion of 3S-vaccinated patients who produced anti-3S Abs in a quantity sufficient to increase their CD4 count and to affect associated immunomodulatory markers. Several studies provide convincing clues that the choice of adjuvant, routes of immunisation and prime/boost strategies could overcome these limitations,^37,38^ and should be tested in future clinical studies to improve the immunogenicity of VAC-3S. Finally, to directly extend the earlier pre-clinical studies performed in SIV-162P3-infected macaques to this human study,^20,21^ analysis of lymphoid tissues should be included in the next clinical trial.

Hence, these encouraging results of VAC-3S not only warrant optimisation of the vaccine strategy but also strongly suggest that combined therapies targeting virus-mediated and NK-mediated CD4 cell protection open new perspectives for fighting the mechanisms of HIV pathogenesis and strengthening the immune status of ART-treated patients.

## Methods

### Study design, approvals, and participants

The IPROTECT1 study was a multicentre, randomised, double-blind, dose-escalating, placebo-controlled phase 2 clinical trial, to assess safety, immunogenicity and effect on CD4^+^ T cells. HIV-1 infected patients aged 18–60 years receiving cART, with plasma HIV RNA levels ≤50 copies/mm^3^ within the past year and a current CD4 count between 200 and 500 cells per mm^3^ were eligible for inclusion. Patients with any immunotherapeutic intervention in the past year, active hepatitis B or C co-infection or any chronic disease other than HIV were not eligible.

The trial was conducted was conducted in clinical centres in France, Spain, and Germany in accordance with Good Clinical Practices and the ethical principles of the Helsinki declaration. The relevant Ethics Review Committees approved the study in France by CPP Ile de France Paris 10, in Germany by the Ethikommission an der Medizinischen Fakultat der Rheinischen Friedrich-Wilhelms-Universitat, and in Spain by the CEIC Hospital Clinic I Provincila de Barcelona. All participants provided written informed consent before entering the study.

In this dose-ranging exploring study, participants were stratified according to their CD4 count (200–349 or 350–500) and allocated to receive three intramuscular injections of VAC-3S or placebo. VAC-3S doses used in this trial were chosen based upon safety and toxicity data from animal studies and early human data from the previous Phase 1 trial, in which a maximum total dose of 200 μg per individual was validated for safety (ClinicalTrials.gov, numbers NCT0159119 and NCT02390466). As a general rule, it was decided to validate each dose escalation via an independent Data Safety Monitoring Board (DSMB). A first group of patients was randomised to receive either VAC-3S 16 μg (Arm A) or 32 μg (Arm B), or placebo (Arm D) every 4 weeks for 3 vaccinations. In absence of observed safety signal following DSMB review, the first 6 subjects in the 32 μg treatment group receive 3 more injections at weeks 20, 32, and 44, to test the clinical relevance of additional maintenance or boosters. Then, the randomisation continued in the arm A and B in addition to a first group of patients of Arm C that received VAC-3S 64 μg doses. In absence of observed safety signal during DSMB review after the first 6 subjects in the 64 μg treatment group were vaccinated, randomisation continued. However, in order to avoid exposing subjects to very high cumulative doses patients of Arm C received only the first three injections (at weeks 0, 4, and 8) (Fig. 2). Furthermore, in order to maintain the double-blind, the 64 µg arm patients received placebo as maintenance vaccinations.

Clinical evaluations were assessed at screening, baseline, 72 h after each injection, and then on a monthly basis up to week 48. Clinical and laboratory events were graded for severity based on the DAIDS toxicity scale (Division of AIDS, National Institutes of Health). The pharmacovigilance was monitoring by Vigipharm (Montpellier, France). Complete laboratory tests, including routine biochemistry tests, blood count, CD4 and CD8 counts, CD4/CD8 ratio, and plasma HIV RNA level were performed weekly.

### Vaccine

VAC-3S (a 16-mer 3 S gp41 HIV-1 peptide)^16^ was synthesised and covalently linked to CRM197 by a linker moiety (Sulfo-SMBP) from PiCHEM (Grambach, Austria). It was formulated with aluminium hydroxide (Alhydrogel, Brenntag Biosector, Frederikssund, Denmark) by GL Pharma (Leuze, Belgium), as a suspension for injection at dose concentrations of 32 and 64 μg/mL. The placebo had the same composition as the study drug with no active substance. Study supplies were produced under current good manufacturing practices.

### Study objectives

The primary endpoint was the proportion of vaccine responders four weeks after the third injection (week 12). The secondary endpoints included the proportion of patients with local and general clinical adverse events, biological tolerability, course over time of plasma HIV viral loads, CD4 counts, CD4/CD8 ratios, and inflammation markers at week 48.

The exploratory endpoints were the change from baseline of immunomodulatory markers in association with a CD4^+^ T cell increased: expression of NKp44L, and cell activation, apoptosis, and exhausted markers.

### Safety analysis

The study team conducted a weekly review of all AEs. The study team remained blinded to treatment assignment for the duration of the study. After reviewing reported events, the study team assessed the relationship of the AE to the study vaccine/placebo injections. An independent data safety monitoring board also reviewed safety data broken down by placebo and vaccine group at 6-month intervals until study completion.

### Determination of VAC-3S IgG antibodies by ELISA assay

Blood samples were collected at baseline (W0), week 4 (W4), W8, W12 (primary end point), W24, W36, and W48 (secondary end point) for more detailed immunologic measurements: Anti-3S Abs were quantified as reported,^18^ using a cut-off of 35 U/mL.

### Quantification of immunological markers

Flow cytometry was used to further characterise CD4^+^ T-cell response to the vaccine with markers for differentiation (CD45Ra, CD27, and CCR7), activation (HLA-DR, CD38), and exhaustion (programmed death 1, PD-1) (Supplementary Table 5). Data were analyzed with Flow Jo version 9 (TreeStar). Inflammatory markers (IL-6, CRP, DDIME, and TNFAR2), Anti-CRM197 Abs and soluble dipeptidylpeptidase 4 (sDPP4) activity were quantified by ELISA assays.

### Functional activity of anti-3S Abs

The capacity of anti-3S Ab to inhibit NKp44L surface expression on CD4^+^ T cells was assessed with 3S peptide-activated CD4^+^ T cells from healthy donors either treated or not for 2 days with serum samples from the different patients (dilution 1:50) and human AB serum as a negative control.^18^ Cell-surface expression of NKp44L was detected by flow cytometry with 1 μg/mL anti-NKp44L mAb (#7.1).^16^

### Intracellular cytokine staining (ICS)

ICS was performed from 10^6^ thawed PBMCs were stimulated with tuberculin purified protein derivate (PPD; 10 μg/mL) (Statens Serum Institute, Copenhagen, Denmark), in 200 μL final volume for 1 h at 37 °C, followed by an additional 5 h incubation in the presence of brefeldin A (Golgi Plug; BD Bioscience). As positive control, cells were stimulated with *Staphylococcus* enterotoxin B (SEB; 2 μg/mL) (Sigma-Aldrich, St. Louis, MI, USA), or media alone as negative control. After cell-surface staining with anti-CD45/CD3/CD4 for 15 min at room temperature, cells were permeabilized (Cytofix/cytoperm; BD Bioscience) at 4 °C and then stained another 15 min for IFN-γ and IL-2, at room temperature before washing and resuspending in PBS until acquisition on Galios flow cytometer (Coulter).^39^

### Statistical analysis

The total sample size of the study to evaluate the primary endpoint was estimated on the assumption of an overall type I error of 2.5%, a one-sided test, with a power of at least 90%. The assumptions regarding responses in the treatment groups were based on the outcome of the phase I study, during which 30% of patients responded to the 10 μg × 3 injections.

The primary end point was analysed by comparing the proportion of immunogenic responders between treatment groups, at week 12. Comparisons were performed by using ANOVA adjusted for the CD4 stratification factor and the baseline value. A responder was defined as a subject with anti-3S Ab titres <35.3 AU at baseline and ≥35.3 AU at week 12 or a subject with anti-3S Ab titres ≥35.3 AU at baseline and a percent change from baseline in anti-3S Ab titres >53.4% at week 12.

Within each group, randomisation to vaccine or placebo took place in two blocks of individuals, in a random order within the block. All investigators, site pharmacists, study nurses, and participants remained blinded until the database was released for statistical analysis.

Patients’ baseline characteristics were summarised by the minima, maxima, medians, means, standard deviations, quartiles, and geometric means (when appropriate) for the quantitative variables, and by frequencies and percentages for the qualitative variables. Comparisons were performed with the Cochran-Mantel-Haenzel (CMH) test, adjusted for the stratification factor (CD4 level at baseline). The closed-testing procedure,^40^ used to handle the multiple testing due to the presence of three doses (16, 32, and 64 µg) to be compared with placebo, enabled us to control the family-wise error rate.

For the primary endpoint, the overall type-I error was set to 2.5%, one-sided. Treatment effects are expressed by the common odds ratio and its 95% confidence interval (CI) and the odds ratio within each stratum with each of their 95% CIs. The 95% CI of responders in each group was also determined, calculated with the Clopper-Pearson method.

Repeated measure analyses of the change from baseline were also performed for anti-3S Abs, CD4 and CD8 counts and percentages, CD4/CD8 ratios, total HIV-DNA levels, and inflammatory markers, with each dose compared to placebo at week 12 and week 48 at the 5% level in a two-sided test. All statistical analyses for the clinical study were performed by Effistat (Paris, France).

URL in registry database: ClinicalTrials.gov, #NCT02041247.

### Reporting summary

Further information on experimental design is available in the Nature Research Reporting Summary linked to this article.

## Supplementary information

Supplementary File 1

Reporting summary

## Data Availability

The data that support the findings of this study are available from the corresponding author upon reasonable request.

## Supplementary information

**Supplementary information** accompanies the paper on the *npj Vaccines* website (10.1038/s41541-019-0117-5).
