# Supplementary material for: HIV therapeutic vaccine enhances non-exhausted CD4+ T cells in a randomised phase 2 trial
Source: NPJ Vaccines. 2019 Jun 3;4:25. doi: 10.1038/s41541-019-0117-5 (PMC6546693; doi:10.1038/s41541-019-0117-5)
Supplement: Supplementary file 1 [file 41541_2019_117_MOESM1_ESM.docx]

**A therapeutic vaccination in HIV-1 patients under suppressive ART to enhance the absolute value of non-exhausted CD4^+^ T cells: a randomised controlled phase 2 clinical study**

#### Vincent Vieillard^1,*^, Béhazine Combadière^1,*^, Roland Tubiana^2,3^, Odile Launay^4^, Gilles Pialoux^5^, Laurent Cotte^6^, Pierre Marie Girard^7^, Anne Simon^2,3^, Yasmine Duboit^2^, Jacques Reynes^8,9^, Jürgen Rockstroh^10^, Felipe Garcia^11^, Jose Gatell^12^, Alain Devidas^13^, Yazdan Yazdanpanah^14^, Laurence Weiss^15,16^, Gerd Fätkenheuer^17,18^, Brigitte Autran^1,2^, Delphine Joyeux^19^, Shahin Gharakhanian^20^, Patrice Debré^1,2^ and Christine Katlama^2,3^

**Supplementary files**

Supplementary-Table 1………………………………….……………………………2

Supplementary-Table 2………………………………………………………….……3

Supplementary-Table 3……………………………………………………….………4

Supplementary-Table 4…………………………………….…………………………5

Supplementary-Table 5…………………………………….…………………………6

Supplementary-Figure 1……………….……...……………..……………………….7

Supplementary- Figure 2………………………………..………..…………………...8

Supplementary-Figure 3……………………………..………..………………………9

Supplementary-Figure 4……………………………..………..……………………...10

Supplementary-Figure 5……………………………..………..……………………...11

Supplementary-Figure 6……………………………..………..……………………...12

**Supplementary-Table 1:** Summary of adverse events

|  | Vaccine group | | | Placebo group  (N=14) |
| --- | --- | --- | --- | --- |
|  | 16 µg  (N=24) | 32 µg  (N=25) | 64 µg  (N=23) |  |
| Treatment emergent adverse events | 23 (95.8%) | 24 (96.0%) | 23 (100.0%) | 13 (92.9%) |
| Treatment emergent adverse events - RELATED | 15 (62.5%) | 20 (80.0%) | 17 (73.9%) | 12 (85.7%) |
| Serious Treatment emergent adverse events | 1 (4.2%) | 3 (12.0%) | 2 (8.7%) | 1 (7.1%) |
| Serious Treatment emergent adverse events –  RELATED | 0 (0.0%) | 0 (0.0%) | 0 (0.0%) | 0 (0.0%) |
| AE leading to premature discontinuation  of study drug | 0 (0.0%) | 1 (4.0%)* | 0 (0.0%) | 1 (7.1%)** |
| AE leading to premature discontinuation  of study drug - RELATED | 0 (0.0%) | 0 (0.0%) | 0 (0.0%) | 0 (0.0%) |

No statistically significant difference has been observed across study arms for any parameter.

* Blood HIV RNA increase

** Acute hepatitis

**Supplementary-Table 2.** Related treatment emergent adverse events by SOC in ≥ 10% patients

|  | Vaccine groups | | | Placebo group  (N=14) |
| --- | --- | --- | --- | --- |
|  | 16 µg  (N=24) | 32 µg  (N=25) | 64 µg  (N=23) |  |
| Patients with any adverse reaction  during the study | 15 (62.5%) | 20 (80.0%) | 17(73.9%) | 12 (85.7%) |
| General disorders and administration site conditions | 14 (58.3%) | 19 (76.0%) | 16 (69.6%) | 11 (78.6%) |
| Infections and infestations | 1 (4.2%) | 2 (8.0%) | 2 (8.7%) | 2 (14.3%) |
| Gastrointestinal disorders | 3 (12.5%) | 3 (12.0%) | 3 (13.0%) | 1 (7.1%) |
| Musculoskeletal and connective tissue disorders | 4 (16.7%) | 5 (20.0%) | 3 (13.0%) | 5 (35.7%) |
| Nervous system disorders | 2 (8.3%) | 3 (12.0%) | 4 (17.4%) | 5 (35.7%) |
| Skin and subcutaneous tissue disorders | 1 (4.2%) | 3 (12.0%) | 2 (8.7%) | 0 (0.0%) |

**Supplementary-Table 3.** Anti-3S antibody production at week 12

|  | Anti-3S antibodies (U/mL); Mean (SD) | | | | |
| --- | --- | --- | --- | --- | --- |
|  | Vaccine group (16 µg) | Vaccine group (32 µg) | Vaccine group (64 µg) | Placebo group | *P values* |
| All patients | | | | | |
| Baseline  Week 12  *p-values* | 21.89 (45.76) | 11.64 (0.70) | 11.81 (1.48) | 17.36 (18.12) |  |
|  | 71.97 (84.47) | 62.43 (67.49) | 62.59 (116.01) | 16.63 (15.30) | ***0.0003*** |
|  | ***0.0026*** | ***0.0002*** | ***0.0020*** | ns |  |
| Strata : CD4 : 200-349 cell/mm^3^ | | | | | |
| Baseline  Week 12 | 27.91 (59.87) | 11.50 (0.00) | 11.50 (0.00) | 23.89 (27.45) |  |
|  | 95.68 (102.33) | 42.11 (22.90) | 33.10 (23.20) | 20.65 (22.41) |  |
|  |  |  |  |  |  |
| Strata : CD4 : 350-500 cell/mm^3^ | | | | | |
| Baseline  Week 12 | 13.46 (6.20) | 11.72 (0.87) | 11.97 (1.83) | 12.46 (2.72) |  |
|  | 38.76 (31.78) | 74.63 (82.21) | 78.32 (141.83) | 13.18 (4.43) |  |
|  |  |  |  |  |  |

**Supplementary-Table 4.** Percentage change over placebo at weeks 12 and 48 in vaccine groups

|  | Vaccine groups | | | | | |
| --- | --- | --- | --- | --- | --- | --- |
|  | At 16 µg  (N=24) | | At 32 µg (N=25) | | At 64 µg (N=23) | |
|  |  | p |  | p |  | P |
| Anti-3S  (AU/mL)  Week 12  Week 48 | 181 (52, 422)  541 (198, 1267) | 0.0018  < 0.0001 | 259 (104, 531)  585 (267, 1179) | <0.0001  <0.0001 | 190 (60, 427)  22 (-4, 54) | 0.0009  0.1030 |
| CD4 count (c/mm^3^)  Week 12  Week 48 | -5.9 (-14.6, 3.7)  2.8 (-9.5, 16.8) | 0.2130  0.6638 | -9.1 (-18.6, 1.5)  1.6 (-10.7, 15.6) | 0.0872  0.8060 | -15.8 (-24.7, -5.7)  -4.8 (-21, 14.5) | 0.0041  0.5882 |
| % CD4  Week 12  Week 48 | 4.28(-3.6, 12.8)  6.9 (-0.5, 14.9) | 0.2858  0.0677 | 1.4 (-3.7, 6.6)  0.8 (-5.5, 7.6) | 0.5914  0.7958 | -0.1 (-5.1, 5.2)  0.1 (-6.4, 8.9) | 0.9722  0.7953 |
| CD8 count (c/mm^3^)  Week 12  Week 48 | -6.6 (-18.0, 6.5)  -0.7 (-15.5, 16.7) | 0.2994  0.9333 | -10.4 (-20.8, 1.4)  1.5 (-11.7, 16.8) | 0.0807  0.8247 | -15.7 (-27.3, -2.3)  -8.4 (-23.3, 9.5) | 0.0245  0.3241 |
| % CD8  Week 12  Week 48 | 1.7 (-2.3, 5.9)  3.3 (-0.8, 7.5) | 0.3926  0.1095 | 0.5 (-3.4, 4.6)  2.0 (-2.9, 7.1) | 0.8089  0.4131 | 0.2 (-3.8, 4.4)  0.5 (-3.4, 4.5) | 0.9213  0.8045 |
| CD4/CD8 ratio  Week 12  Week 48 | 1.6 (-8.6, 12.9)  4.3 (-5.0, 14.6) | 0.7683  0.3655 | 2.4 (-4.5, 9.7)  1.4 (-6.4, 9.8) | 0.5003  0.7241 | -0.06 (-7.5, 8.0)  1.99 (-6.4, 11.2) | 0.9872  0.6431 |
| HIV DNA  (c/10^6^ PBMC)  Week 12  Week 48 | -1.0 (-29.0, 38.1)  -15.3 (-44.4, 28.9) | 0.9508  0.4249 | -12.3 (-38.4, 24.8)  -30.9 (-57.5, 12.2) | 0.4524  0.1289 | -2.7 (-28.2, 31.9)  -11.8 (-41.7, 33.6) | 0.8562  0.5419 |
| HIV RNA  (c/mL)  Week 12  Week 48 | 25.7 (-60.3, 298.1)  16.5 (-60.4, 242.8) | 0.6887  0.7752 | -26.9 (-75.5, 118.5)  11.8 (-69.0, 302,9) | 0.5638  0.8605 | 27.7 (-61.8, 326.3)  -39.5 (-78.9, 73.7) | 0.6827  0.3374 |
| IL-6 (pg/mL)  Week 12  Week 48 | 7.8 (-35.9, 81.3)  -27.6 (-55.5, 17.8) | 0.7716  0.1863 | -17.9 (-55.0, 49.7)  -7.6 (-44.7, 54.3) | 0.5091  0.7553 | -14.9 (-51.4, 48.9)  16.0 (-36.2, 111.1) | 0.5613  0.6168 |
| CRP (mg/mL)  Week 12  Week 48 | 18.2 (-42.5,143.2)  13.7 (-41.1, 119.6) | 0.6398  0.6940 | 59.9 (-17.0, 208.1)  71.6 (-7.1, 216.9) | 0.1547  0.0827 | 12.7 (-45.8, 134.3)  18.3 (-41.1, 137.4) | 0.7418  0.6269 |
| DDIMER  (pg/mL)  Week 12  Week 48 | 21.10 (-22.9, 90.2)  46.6 (0.3, 114.2) | 0.3929  0.0485 | -2.77 (-33.5, 42.2)  25.8 (-18.5, 94.1) | 0.8812  0.2895 | -10.7 (-41.9, 37.2)  -10.3 (-40.0, 34.2) | 0.5948  0.5849 |
| TNFAR2  (pg/mL)  Week 12  Week 48 | 0.8 (-10.4, 13.4)  0.1 (-13.5, 15.8) | 0.8908  0.9928 | -4.5 (-15.8, 8.3)  -1.0 (-14.7, 15.0) | 0.4617  0.8958 | 0.2 (-11.8, 13.8)  4.2 (-12.1, 23.6) | 0.9766  0.6225 |

**Supplementary-Table 5.** List of commercial antibodies used in the study

| **Marker** | **Clone** | **Company** | **Catalog number** |
| --- | --- | --- | --- |
| CD45 | J.33 | Coulter | A96416 |
| CD3 | UCHT1 | Coulter | B10823 |
| CD4 | 13B8.2 | Coulter | IM0448U |
| CD8 | RPA-T8 | BD pharmingen | 557746 |
| HLA-DR | L243 | BioLegend | 307605 |
| CD38 | LS198-4-3 | Coulter | A99022 |
| CD45Ra | 2H4 | Coulter | B10821 |
| CD27 | 1A4CD27 | Coulter | B12701 |
| CCR7 | G043H7 | Coulter | B30632 |
| PD1 | PD1.3 | Coulter | B30634 |
| IFN-γ | B27 | BioLegend | 506510 |
| IL-2 | N7.48A | Coulter | IM2718U |


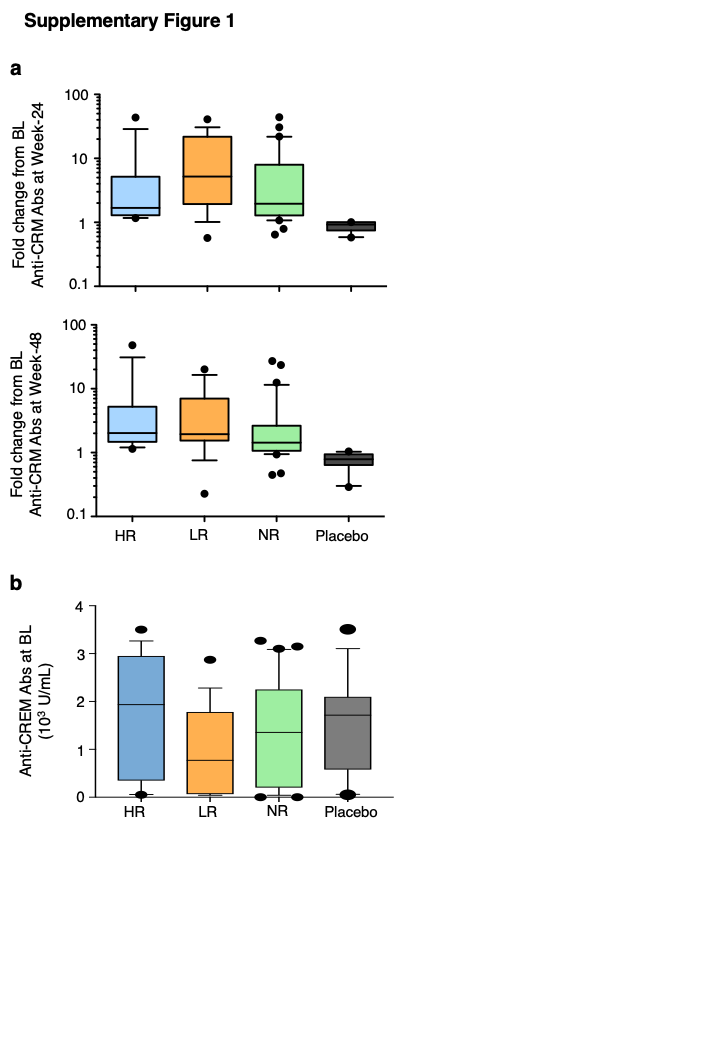


**Supplementary-Figure 1.** Production of anti-CREM Abs in all serum samples from high (HR), low (LR), and non-responders (NR), compared to placebo. Fold changes of anti-CRM Ab level from baseline (BL) to week 24 and week 48 in **a**. Production of anti-CREM Ab level at baseline (BL) in **b**. Data are expressed as Unit of Abs /per mL (U/mL). Horizontal bars represent the median.


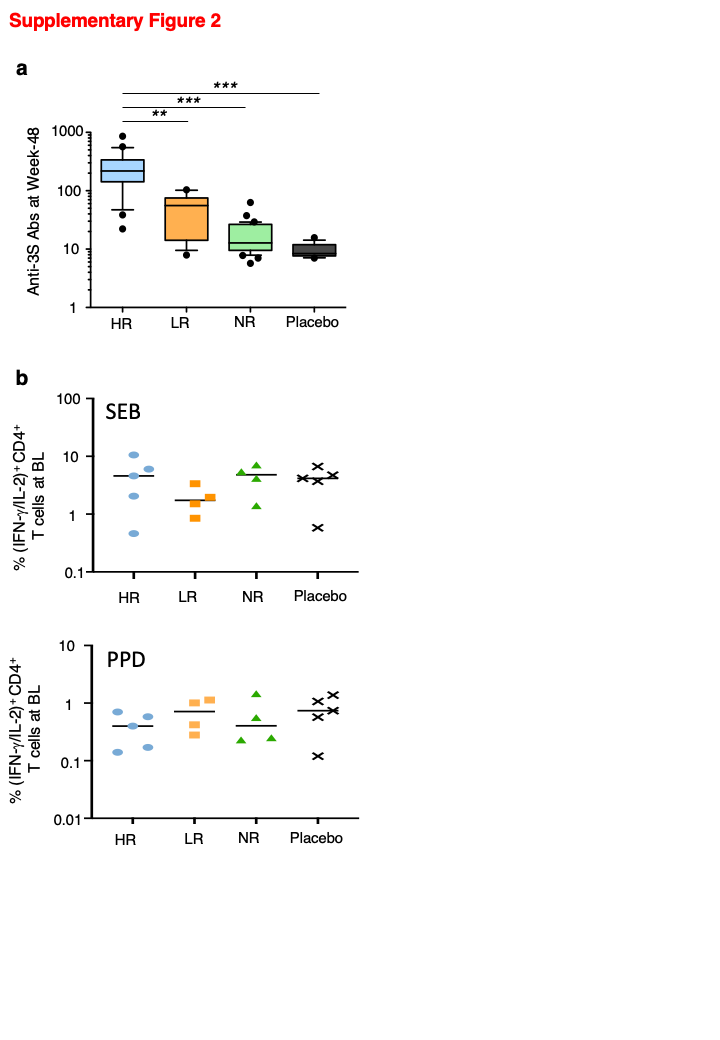


**Supplementary-Figure 2.** Anti-3S Ab production, in **a**. Serum samples from high (HR), low (LR), and non-responders (NR), compared to placebo were quantified at week 48 in serum samples. Data are expressed as unit per mL (U/mL). **: p<0.001; ***: p<0.0001 (Mann-Whitney test). CD4^+^ T cell responses to SEB and PPD at the baseline (BL) in **b**. Experiments were assessed after peripheral blood mononuclear cells (PBMC) stimulation with [*Staphylococcus*](https://www.sciencedirect.com/topics/immunology-and-microbiology/staphylococcus) [enterotoxin](https://www.sciencedirect.com/topics/immunology-and-microbiology/enterotoxin) B (SEB, 2 μg/mL) or PPD (10 μg/mL) at baseline (BL) in high (HR, n=5), low (LR, n=4), and non-responders (NR, n=4), compared to placebo (n=5). All values have been corrected for isotype controls and production in the absence of recall Ag. Horizontal bars represent the median.


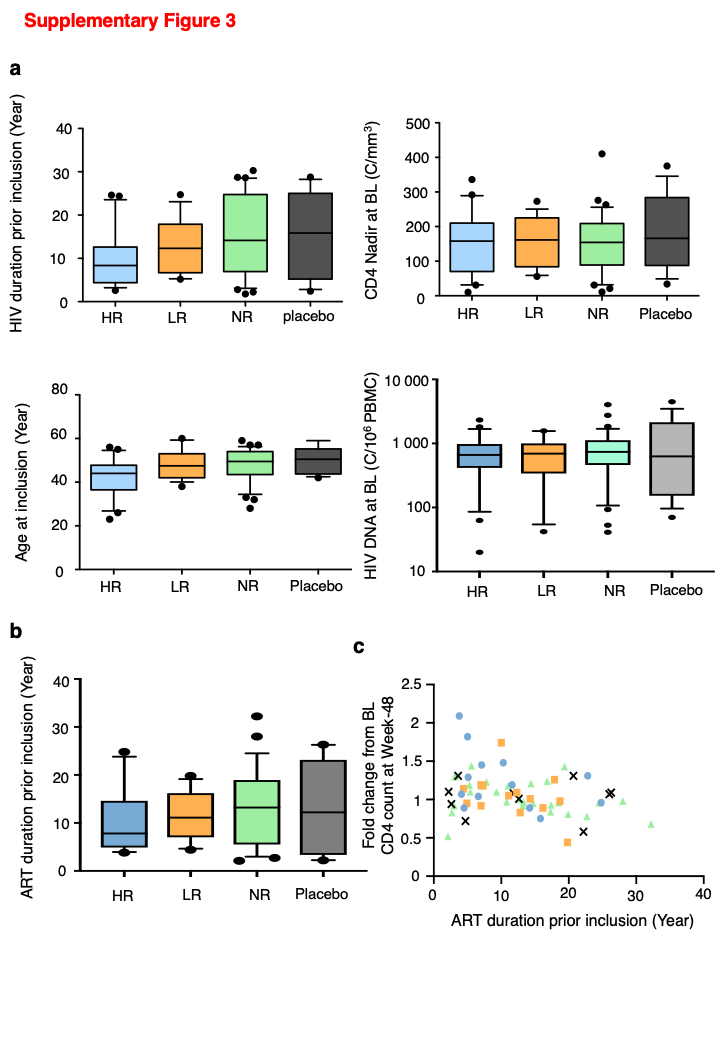


**Supplementary-Figure 3.** Other clinical parameters. HIV duration before inclusion, CD4 nadir and HIV DNA at baseline (BL), as well as age at inclusion in high (HR), low (LR), and non-responders (NR), compared to placebo, in **a**. ART duration prior inclusion in **b**. Correlation between ART duration prior inclusion and fold-change from BL of CD4 count at week 36 in **c,** from high (HR, blue circles), low (LR, orange squares), and non-responders (NR, green triangles), compared to placebo (grey cross).


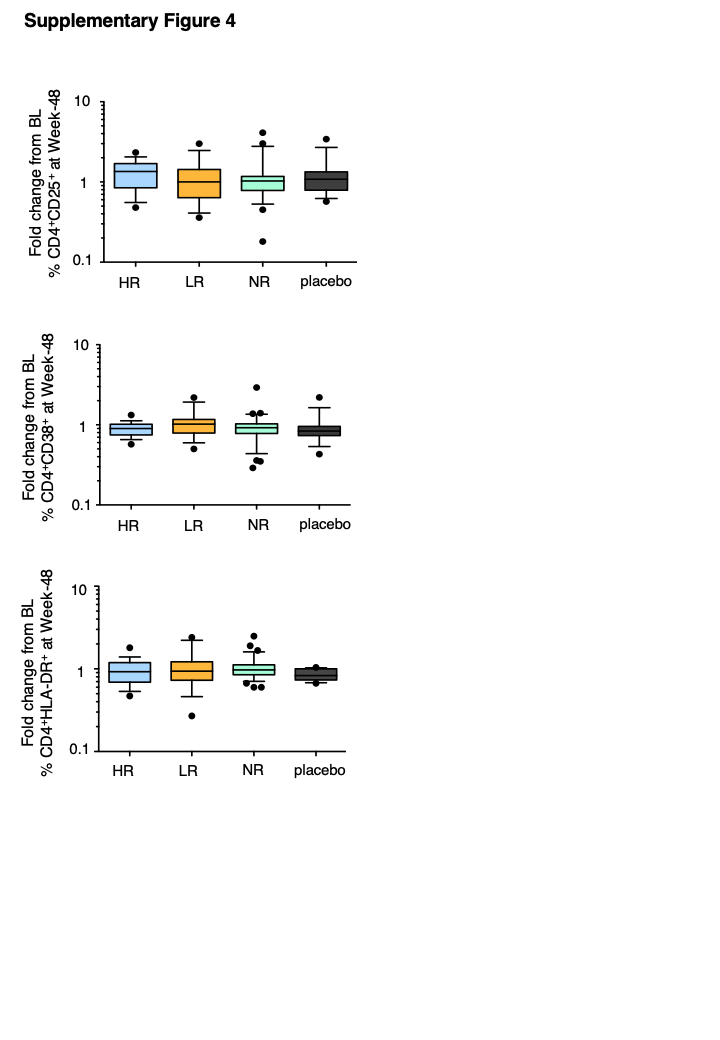


**Supplementary-Figure 4.** Fold changes of cell-activation markers on CD4^+^ T cells. Samples were tested from baseline (BL) to week 24 and week 48 in serum samples from high (HR), low (LR), and non-responders (NR), compared to placebo. Horizontal bars represent the median.


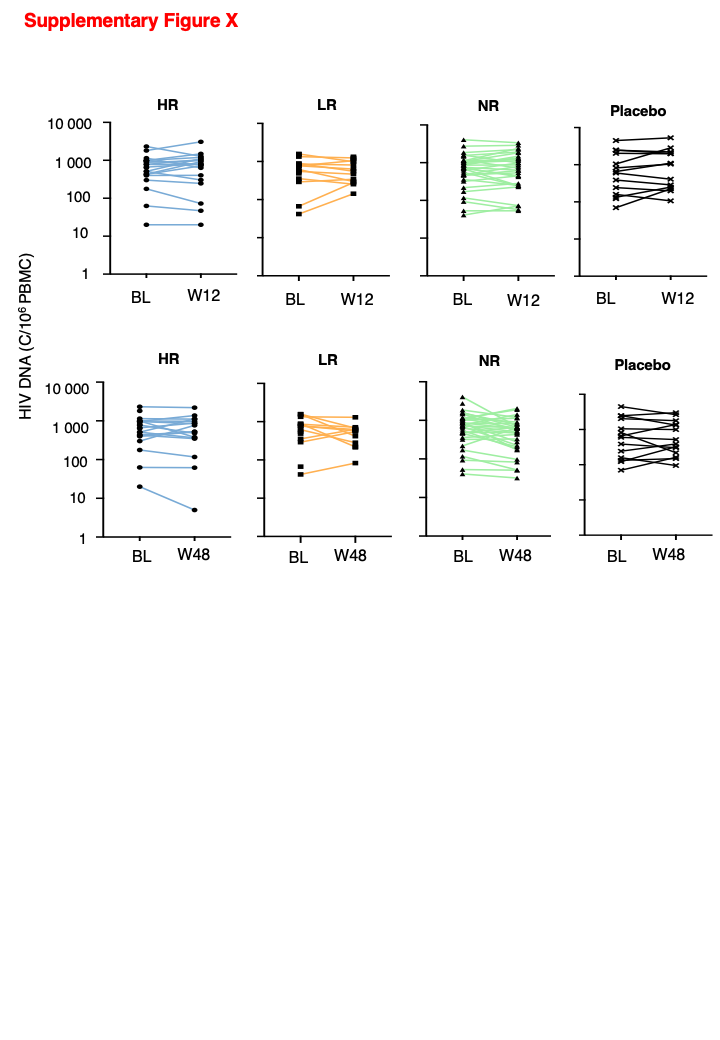


**Supplementary-Figure 5.** Course of HIV DNA between baseline (BL) and week 12 (W12, upper panels) or week 48 (W48, low panels) in high (HR), low (LR), non-responders (NR), and placebo. C/10^6^ PBMC: copies per 10^6^ PBMC.


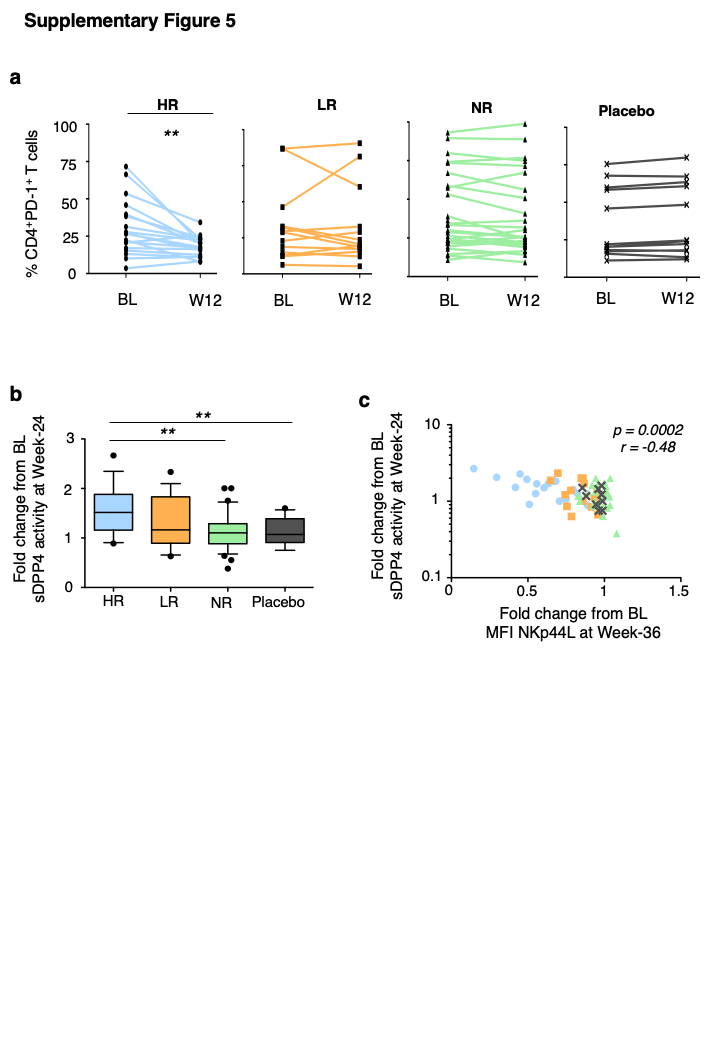


**Supplementary-Figure 6.** Supplementary immunomodulatory data**.** Course of PD-1 expression on CD4^+^ T cells between baseline (BL) and week 12 (W12) in high (HR), low (LR), non-responders (NR), and placebo in **a**. **: p<0.001 (Wilcoxon matched-pairs test). Fold changes from baseline to week 24 of sDDP4 activity in serum samples from high (HR), low (LR), and non-responders (NR), compared to placebo in **b**. Horizontal bars represent the median. **: p<0.001. Correlation of changes from baseline (BL) between sDDP4 activity in serum samples at week 24 and NKp44L inhibition at week 36, expressed in mean of fluorescence intensity (MFI) on CD4^+^ T cells treated by serum samples in **c** from high (HR), low (LR), and non-responders (NR), compared to placebo. High responders (blue circles), low responders (orange squares), non-responders (green triangles), and placebo (grey cross). Correlation was evaluated using a Spearman rank correlation coefficient test.
